# Population Pharmacokinetic Modelling and Simulation to Determine the Optimal Dose of Nanoparticulated Sorafenib to the Reference Sorafenib

**DOI:** 10.3390/pharmaceutics13050629

**Published:** 2021-04-28

**Authors:** Ki-Young Huh, Se-jung Hwang, Sang-Yeob Park, Hye-Jung Lim, Mir-yung Jin, Jae-seong Oh, Kyung-Sang Yu, Jae-Yong Chung

**Affiliations:** 1 Department of Clinical Pharmacology and Therapeutics, Seoul National University College of Medicine and Hospital, Seoul National University, Seoul 03080, Korea; zealot648@snu.ac.kr (K.-Y.H.); hsejung@snu.ac.kr (S.-j.H.); johan25@snu.ac.kr (J.-s.O.); ksyu@snu.ac.kr (K.-S.Y.); 2 Samyang Biopharmaceuticals Corp., Gyeonggi-do 13488, Korea; sangyeob.park@samyang.com (S.-Y.P.); hyejung.lim@samyang.com (H.-J.L.); miryung.jin@samyang.com (M.-y.J.); 3 Department of Clinical Pharmacology and Therapeutics, Seoul National University College of Medicine and Bundang Hospital, Gyeonggi-do 13620, Korea

**Keywords:** sorafenib, pharmacokinetics, bioavailability, pharmacometrics, enterohepatic reabsorption

## Abstract

Sorafenib, an oral multikinase inhibitor, exhibits a highly variable absorption profile due to enterohepatic reabsorption and poor solubility. SYO-1644 improved the solubility of sorafenib by nanoparticulation technology leading to enhanced bioavailability. To evaluate the pharmacokinetically equivalent dose of SYO-1644 to the reference Nexavar^®^ 200 mg, a randomized, open-label, replicated two-period study was conducted in healthy volunteers. A total of 32 subjects orally received a single dose of the following assigned treatment under a fasted state in the first period and repeated once more in the second period with a two-week washout: SYO-1644 100, 150 and 200 mg and Nexavar^®^ 200 mg. Pharmacokinetic (PK) samples were collected up to 168 h post-dose. The PK profile was evaluated by both non-compartmental analysis and population PK method. With the final model, 2 × 2 crossover trial scenarios with Nexavar^®^ 200 mg and each dose of SYO-1644 ranging from 100 to 150 mg were repeated 500 times by Monte Carlo simulation, and the proportion of bioequivalence achievement was assessed. Transit absorption compartments, followed by a one-compartment model with first-order elimination and enterohepatic reabsorption components were selected as the final model. The simulation results demonstrated that the SYO-1644 dose between 120 and 125 mg could yielded the highest proportion of bioequivalence.

## 1. Introduction

Sorafenib is an oral multikinase inhibitor approved for the treatment of advanced renal cell carcinoma, hepatocellular carcinoma and thyroid carcinoma [1]. Sorafenib targets both downstream Raf serine/threonine kinases and cell surface receptor tyrosine kinases including vascular endothelial growth factor receptors [2,3]. Sorafenib has shown dose-dependent antitumor activity in murine xenograft models [2]. The exposure–response relationship of sorafenib has been confirmed in several clinical trials [4,5]. 

Sorafenib exhibits large inter-individual variability in pharmacokinetics (PK) owing to its poor solubility [6]. The solubility of sorafenib ranges from 0.034 to 0.013 mg/100 mL, classified as a Biopharmaceutics Classification System II (low solubility and high permeability) drug [7]. The large variability has been suggested as a source of treatment failure or excessive toxicities of sorafenib [4,8,9]. 

SYO-1644 is a sorafenib-loaded nanoparticle formed by applying Nanoparticulation Using Fat and Supercritical Fluid (NUFS^TM^) technology to improve the solubility. [10]. A higher solubility profile was achieved for SYO-1644 by optimizing the particle size and dissolution profile using design space [10]. This was confirmed in a pharmacokinetic study in beagle dogs [10]. SYO-1644 had an approximately 1.8-fold increase in systemic exposure compared to the reference, Nexavar^®^ [10]. Therefore, at a lower dose, SYO-1644 was expected to show equivalent systemic exposure to Nexavar^®^.

However, the complex PK profile of sorafenib requires much consideration for determining the SYO-1644 dose equivalent to Nexavar^®^ (specifically for the approved 200 mg). The absorption PK profile of sorafenib is characterized by multiple irregular peaks [9,11]. This makes it difficult to predict the maximum concentration (C_max_) and area under the time-concentration curve (AUCs) of sorafenib with a simple PK model, which are two crucial elements for demonstrating bioequivalence [12]. Similar complications have been addressed in highly variable drugs [13,14], such as piroxicam [15]. These intrinsic PK characteristics significantly interfere with the evaluation of formulations, reducing the statistical power of the study [14].

To determine the equivalent dose of SYO-1644 to Nexavar^®^ 200 mg, a randomized, open-label, replicated two-period study was conducted in healthy volunteers. Based on the results of the PK study, a population PK model was developed, and simulations for bioequivalence studies were conducted to estimate the optimal equivalent dose of SYO-1644. 

## 2. Materials and Methods

### 2.1. Study Subjects 

Healthy male subjects were enrolled aged 19–50 years with a body mass index (BMI) between 18.0 and 27.0 kg/m^2^. Subjects were evaluated for eligibility criteria by medical interviews, vital signs (systolic and diastolic blood pressure, pulse rate, and body temperature), 12-lead electrocardiograms, physical examinations, and clinical laboratory tests. Subjects with clinically significant disease or medical history were excluded. Written informed consent form was obtained from all subjects prior to any study-related procedures. The study was approved by the Institutional Review Board of Seoul National University Hospital (Clinicaltrials.gov registration no. NCT03674060, date of approval: 20 July 2018) and conducted in accordance with the Declaration of Helsinki.

### 2.2. Study Design

**Figure 1:**
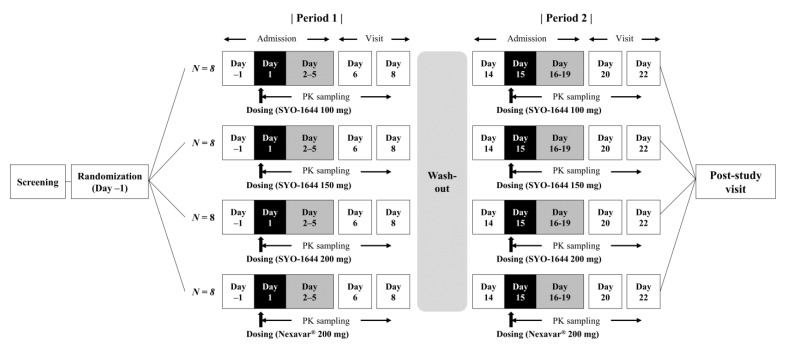
Study scheme.

A randomized, open-label, replicated two-period study was conducted (Figure 1). Subjects were randomly assigned to one of the following treatments: SYO-1644 100, 150, or 200 mg or Nexavar^®^ 200 mg. Subjects orally received a single dose of the assigned treatment in a fasted state during the first period and repeated once more during the second period with a two-week washout. Blood PK samples for sorafenib were collected at pre-dose and 0.5, 1, 2, 3, 4, 6, 8, 10, 12, 24, 36, 48, 72, 96, 120, and 168 h post-dose in each period. Meals were provided approximately 4 and 10 h after the PK sample was collected.

### 2.3. Bioanalytical Method

Blood PK samples for sorafenib were collected in sodium heparin tubes and centrifuged at 2000× *g* at 4 °C for 10 min. The supernatant was separated and stored below 70 °C until the assay. The samples were prepared by protein precipitation using acetonitrile. Sorafenib-D3 was used as an internal standard. The plasma concentrations of sorafenib were determined by validated liquid chromatography (Agilent 1260 Infinity System, Agilent Technologies, Santa Clara, CA, USA) [11]. Chromatographic separation was performed using a Luna C18 column (2.0 mm × 100 mm, μm particle size; Phenomenex, Torrance, CA, USA) at a flow rate of 0.35 mL/min. Mass spectrometry was performed in positive electrospray ionization mode using an API4000 (AB SCIEX, Framingham, MA, USA). The ion transition was monitored at 465.155 → 270.100 *m/z* for sorafenib and at 468.094 → 273.200 *m/z* for sorafenib-D3. The lower limit of quantification for the plasma assay was 5 ng/mL. The calibration curves showed linearity in the range of 5–5000 ng/mL. The overall accuracy of the calibration curve was 92.6–106.9%, and the precision was within 4.3%.

### 2.4. Noncompartmental Pharmacokinetic Analysis

PK parameters for sorafenib were initially calculated by non-compartmental analysis using Phoenix^®^ WinNonlin^®^ (version 7.0; Certara USA, Princeton, NJ, USA). The C_max_ and time to reach C_max_ (T_max_) were directly obtained from the observed values. The AUC from dosing to the last measurable concentration (AUC_last_) was calculated using the linear trapezoidal method for ascending concentrations and the log trapezoidal method for descending concentrations. The terminal elimination constant was estimated using linear regression. The terminal elimination half-life (t_1/2_) was calculated as the natural logarithm of the two divided by the estimated terminal elimination rate constant. The log-transformed C_max_ and AUC_last_ of each treatment group were analyzed using the linear mixed effect model that included the treatment group and period as fixed effects and the subject as the random effect. The intrasubject coefficient of variation (CV_intra_) of each treatment was calculated from the residual error in the linear mixed-effect model using the following Equation (1):

(1) $${\mathrm{CV}}_{\mathrm{intra}}\left(\%\right)=100\cdot \sqrt{\exp\left(\omega^{2}\right)-1}$$

ω: residual error in the linear mixed effect model.

### 2.5. Development of the Population PK Model

The population PK model was developed using the nonlinear mixed effect modeling (NONMEM) software version 7.4.4. (ICON Development Solutions, Ellicott City, MD, USA). The estimation procedure was carried out using first-order conditional estimation with the interaction (FOCE-I) option. Data processing and diagnostics were conducted using R (version 3.6.3; R Core Team, Vienna, Austria), Xpose version 4 [16], and Perl-speaks-NONMEM 4.9.0. [17].

The PK profiles of sorafenib showed enterohepatic reabsorption characterized by multiple peaks [2]. The proposed structure models for enterohepatic reabsorption were evaluated: one- and two-compartment models with absorption lag time, combined absorption of first- and zero-order kinetics, gall bladder-based models with continuous release, switch function, or sigmoid function [18,19,20]. The relative bioavailability of SYO-1644 to Nexavar^®^ was parameterized for evaluation. Meal times (i.e., lunch and dinner) at the dosing date were also included as parameters.

Inter-individual variability (IIV) and inter-occasional variability (IOV) were described using an exponential model. The IOV was modeled using the OMEGA BLOCK step in NONMEM [21]. A combined proportional and additive residual error model was used to describe the random error in plasma concentrations [22]. 

### 2.6. Evaluation of the Final Population Pharmacokinetic Model 

Goodness-of-fit plots of individual/population observed versus predicted concentrations, conditional weighted residuals (CWRES) versus population predictions and CWRES versus time after dose were graphically evaluated by each model. Visual predictive check (VPC) and bootstrapping were conducted to evaluate the predictive performance and stability of the model. VPC was conducted with 200 simulated sample subjects, and plasma concentration–time profiles were stratified by treatment group (SYO-1644 100, 150, 200 mg or Nexavar^®^). Bootstrapping was conducted with 2000 replicates to calculate the median and 95% confidence intervals for the population PK model parameter estimates. 

### 2.7. Simulation of Bioequivalence Trials

With the final model, 2 × 2 crossover trial scenarios with Nexavar^®^ 200 mg and each dose of SYO-1644 ranging from 100 to150 mg (5 mg interval) and the number of subjects ranging from 24 to 48 subjects (4 subjects interval) were repeated 500 times by Monte Carlo simulation. PK time points for the simulated trial were the same as those used in the study. C_max_ and AUC_last_ in each simulated trial were calculated by noncompartmental analysis using the *NonCompart* R package [23]. Achievement of bioequivalence was defined as when the 90% CI was within 0.8–1.25 and was evaluated separately for C_max_ and AUC_last_. The sample proportion of bioequivalence achievement ($\hat{p}$) in the 500 simulated bioequivalence trials was utilized to estimate the 95% confidence intervals for the population proportion (p). The 95% confidence interval was estimated on the asymptotic normality of the sample proportion using the following equation were estimated with the following Equation (2) [24]:

(2) $$p=\hat{p}\pm Z_{\alpha /2}\;\sqrt{\frac{\hat{p}\cdot \left(1-\hat{p}\right)\;}{n}}$$

$Z_{\alpha /2}:1-\alpha /2$ quantile of the standard normal distribution; α: significance level (=0.05); n: the number of simulated trials (=500).

### 2.8. Safety and Tolerability Assessments

Safety and tolerability were assessed by adverse event monitoring, physical examinations, vital signs, 12-lead electrocardiograms and clinical laboratory tests.

### 2.9. Statistical Analysis

Baseline demographics were summarized using descriptive statistics and compared between treatment groups using the Kruskal–Wallis test. The likelihood ratio test was performed between the two nested models. A decrease in the objective function value greater than 3.84 was considered a statistically significant improvement of the model when a parameter was included in the model [25].

## 3. Results

### 3.1. Subject Disposition and Demographics

A total of 32 subjects were enrolled, and 30 subjects completed the study. Two subjects (one in SYO-1644 100 mg and another in the 150 mg group) withdrew their consent after completing the first period. For noncompartmental analysis, 30 subjects who completed the study were included, and for the development of the population PK model, the 32 subjects who had at least one measurable PK concentration were included. There were no significant differences in demographics between the treatments. Mean and standard deviation (*p*-value for Kruskal–Wallis test between treatments) of age, height, weight and body mass index of the enrolled subjects were 30.9 ± 7.7 (years, *p* = 0.6801), 173.8 ± 5.0 (cm, *p* = 0.5503), 71.0 ± 8.2 (kg, *p* = 0.9786) and 23.5 ± 2.4 (kg/m^2^, *p* = 0.9729), respectively.

### 3.2. PK Profiles and Noncompartmental Analysis Results

**Figure 2:**
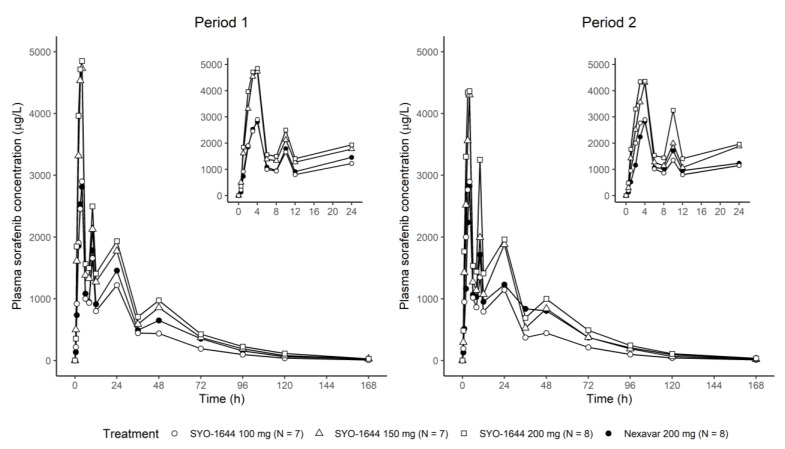
Mean plasma sorafenib concentration after single administration of SYO-1644 100, 150, 200 mg and Nexavar^®^ 200 mg (inset: time points until 24 h).

Sorafenib was absorbed irregularly and showed multiple peaks (Figure 2). The maximum concentration was reached after 4 h post-dose, and the second and third peaks were observed at 10 and 24 h post-dose, commonly in all groups. The terminal half-life ranged from 20 to 25 h and was similar between the groups. Nexavar^®^ showed a lower total CV in C_max_ than every dose of SYO-1644 except for total CV for SYO-1644 200 mg. In contrast, Nexavar^®^ showed a higher total CV of AUC_last_ than every dose of SYO-1644. The same dose of SYO-1644 showed approximately 1.5-fold greater AUC_last_ than the reference 200 mg (Table 1).

### 3.3. Population Pharmacokinetic Analysis

Transit absorption compartments, followed by a one-compartment model with first-order elimination and enterohepatic reabsorption components were selected as the final model. (Table S1) Relative bioavailability (SYO-1644 to Nexavar^®^) was estimated to be 1.62. The transit absorption compartments were parameterized with the mean transit time (MTT), number of transit compartments (NN), and absorption rate constant (k_a_). MTT and the NN were estimated as 3.24 h and 1.72, respectively; k_a_ was estimated as 1.6 h^−1^ and showed a high intersubject CV of 87.3%. Volume of distribution (V) and clearance (CL) in the central compartment were estimated as 8.17 L and 3.33 L/h. IIV on V and CL improved the fit of the model and was estimated to be 4.5% and 6.6%, respectively. Only IOV on CL improved the fit of the model and was estimated to be 8.9% (Table 2).

**Figure 3:**
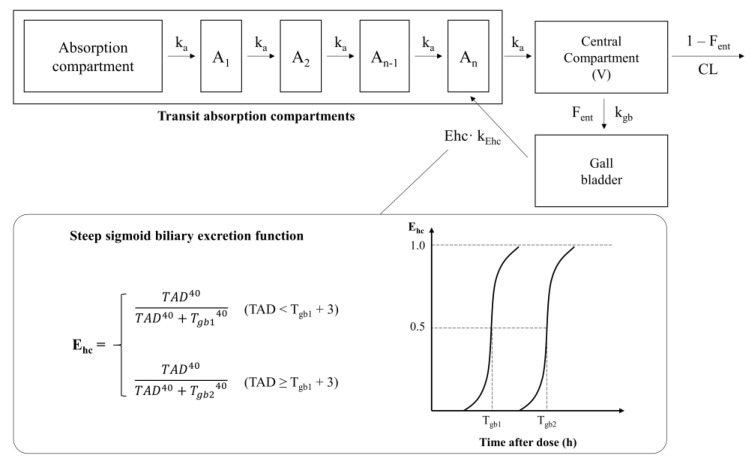
Structure representation of the final model. (k_a_, absorption rate constants; CL, clearance of the central compartment; V, volume of distribution of the central compartment; F_ent_, fraction of enterohepatic reabsorption; k_Ehc_, enterohepatic reabsorption constant; T_gb1_, early gall bladder emptying time; T_gb2_, late gall bladder emptying time).

The enterohepatic reabsorption components consisted of the gall bladder compartment combined with a steep-sloped sigmoid biliary excretion function proposed by Jain et al. [9] (Figure 3). The gall bladder compartment was linked to the central compartment with first-order transfer multiplied by the fraction of the enterohepatic compartment (F_ent_). First-order secretion of bile into the absorption compartment was parameterized with the enterohepatic reabsorption constant (k_Ehc_) and bile steep-sloped sigmoidal function (E_hc_). To describe the multiple peaks observed during the absorption of sorafenib, E_hc_ was modified with the following two parameters: early (T_gb1_) and late (T_gb2_) gall bladder emptying time. E_hc_ was assumed to be reset when 3 h elapsed from Tg_b1_. The slope of the sigmoid function was set to 40, which was similar to the previous model [9]. E_hc_ was defined using the following Equation (3):

(3) $$E_{\mathrm{hc}}=\frac{{\mathrm{TAD}}^{40}}{{\mathrm{TAD}}^{40}+T_{\mathrm{gb}1}{}^{40}}\;\mathrm{when}\;\mathrm{TAD}\;<\;T{\mathrm{gb}}_{1}+3\;\frac{{\mathrm{TAD}}^{40}}{{\mathrm{TAD}}^{40}+T_{\mathrm{gb}2}{}^{40}}\;\mathrm{when}\;\mathrm{TAD}\;\geq \;T{\mathrm{gb}}_{1}+3$$

TAD: time after dose

Based on the newly defined E_hc_, T_gb1_ and T_gb2_ were estimated to be approximately 8 and 12 h post-dose, respectively (Figure 3, Table 2). 

**Figure 4:**
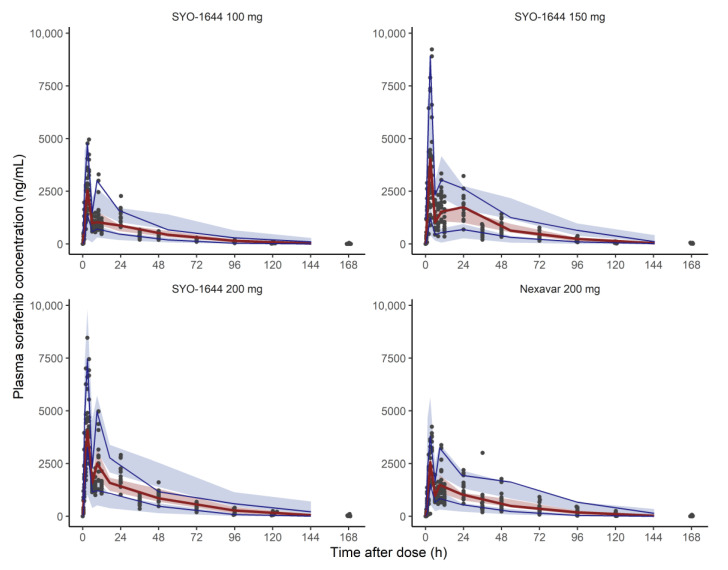
Visual predictive check results for the final population pharmacokinetic model. Solid blue lines represent 5th and 95th percentile for observed data. Solid red line represents median for observed data. Blue areas represent 95% confidence intervals for 5th and 95th percentile of simulated data. Red areas represent 95% confidence intervals for median of simulated data.

The goodness-of-fit plots showed that the model predicted concentrations that showed a reasonable correlation with the observed concentrations. No systemic deviations in CWRES were observed (Supplementary Materials Figure S1). The VPC results showed that the model reasonably predicted the observed values for SYO-1644 100, 150 and 200 mg and Nexavar^®^ 200 mg (Figure 4). All parameter estimates in the final model were within the 95% confidence intervals obtained by bootstrapping (Table 2).

### 3.4. Simulation Results for the Bioequivalence Trials 

**Figure 5:**
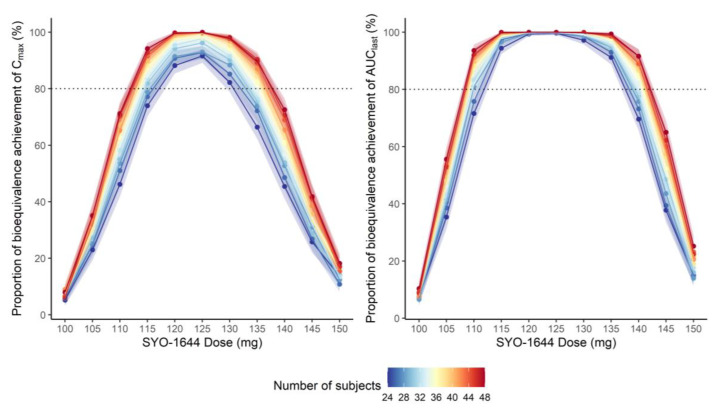
Proportion of bioequivalence achievement (left: C_max_, right: AUC_last_) from simulated 2 × 2 bioequivalence trials for 500 times according to SYO-1644 dose and the number of subjects in each trial. Points represent proportion of bioequivalence achievement and areas represent 95% confidence intervals for proportion of bioequivalence achievement.

The simulation results demonstrated that a SYO-1644 dose between 120 and 125 mg yielded the highest proportion of bioequivalence achievement with Nexavar^®^ 200 mg for both C_max_ and AUC_last_. The proportion of bioequivalence achievement was lower for C_max_ than for AUC_last_ (Figure 5, Table S2). The mean intrasubject CV for C_max_ was 22.0%, and AUC_last_ was 15.7% in the simulated crossover trials (Table S3).

### 3.5. Safety and Tolerability

No serious adverse events or discontinuation due to adverse events were reported. A total of 13 treatment-emergent adverse events in 7 subjects and 6 adverse drug reactions in 2 subjects were reported. All adverse events were mild in severity and recovered without sequelae. No clinically significant differences were noted in the frequency and characteristics of the adverse events between treatments.

## 4. Discussion

SYO-1644 showed a 1.5-fold greater AUC than Nexavar^®^ at the same dose level. The AUC of SYO-1644 increased in a dose-proportional manner. Both SYO-1644 and Nexavar^®^ reached T_max_ 4 h post-dose followed by multiple peaks at 10 and 24 h post-dose. The multiple peaks were well described by the enterohepatic reabsorption model proposed in previous literature with some modifications [9]. The simulation results indicated that SYO-1644 120 or 125 mg could be the pharmacokinetically equivalent dose to Nexavar^®^ 200 mg.

There were significant differences in the parameter estimates compared to those estimated from solid tumor patients [9]. The parameter estimates for CL and V in our model (3.33 L/h and 8.2 L) were smaller to those reported in the solid tumor patients (8.13 L/h and 213 L) [9]. The F_ent_ was also estimated to be smaller than the patient counterpart (0.215 versus 0.498) [9]. A difference in the patient population and dosing scheme could be a possible source of this difference. In a previous study, patients with solid tumor received 200 or 400 mg of sorafenib twice daily [9]. In contrast, in our study, healthy volunteers received a single dose of sorafenib. Furthermore, the time-dependent increase in the clearance of sorafenib [5,26] also needs to be considered as patients with solid tumor who received multiple oral doses of sorafenib. 

Our model introduced two gall bladder emptying times (T_gb1_ and T_gb2_), reflecting the relatively regular meal times in our study. The introduction of the two parameters facilitated the description of peaks observed between 8 and 12 h post-dose, which appeared irrespective of the formulations. The first gall bladder emptying (t_gb_) started approximately 2 h later (8 h vs. 6 h post-dose). Our approach was able to incorporate the steep-sloped bile secretion model of sorafenib [9] and periodic bile secretion during meal times the in the ezetimibe model [27]. 

The relative bioavailability estimated from the population PK model was similar to that estimated from the non-compartmental analysis (1.72 for C_max_ and 1.51 for AUC_last_, respectively). As the relative bioavailability was estimated differently for C_max_ and AUC_last_, a simulation approach was adopted to coordinate both PK parameters. The simulation results showed that a SYO-1644 dose of 120–125 mg was able to achieve bioequivalence not only for AUC_last_ but also for C_max_ with 32–36 subjects (statistical power > 80%). However, sample sizes need to be set more conservatively as the intrasubject CVs reported in our simulation were slightly low.

This study was the first to apply a population pharmacokinetic model to design an optimal bioequivalence trial of sorafenib in healthy volunteers. In our study, we found that the intra-subject CV of sorafenib was not always larger than 30%, which did not favor the replicated design to evaluate bioequivalence [28]. Based on this notion, we selected conventional 2 × 2 crossover trials for the simulation [29].

Our study has some limitations. This study was conducted with a small number of healthy subjects. This might limit the application of these results to the patient population. The absence of sampling points between 12 and 18 h limited a more precise specification of the multiple peak profiles in the population model. However, considering that typical bioavailability/bioequivalence studies are recommended in healthy subjects, the results of our study could be extrapolated to these studies. Furthermore, PK modeling and simulation supported the determination of the optimal study design and dose of SYO-1644 in a more precise manner.

The results of our study can be utilized for future comparative PK studies. From the perspective of drug development, SYO-1644 is needed to show equivalent safety and efficacy profiles to the reference Nexavar^®^. Demonstration of pharmacokinetic equivalence could be a plausible strategy, although the exposure-response relationship must be considered.

## 5. Conclusions

The results of our study confirmed the enhanced bioavailability of SYO-1644. Transit absorption compartments, followed by a one-compartment model with first-order elimination and enterohepatic reabsorption components properly describe the PK profiles of sorafenib. A SYO-1644 dose of 120–125 mg would be the equivalent dose to Nexavar^®^ 200 mg.

## Figures and Tables

**Table 1:** Summary of pharmacokinetic parameters of sorafenib.

Parameters ^†^	SYO-1644 100 mg (*N* = 7)	SYO-1644 150 mg (*N* = 7)	SYO-1644 200 mg (*N* = 8)	Nexavar^®^ 200 mg (*N* = 8)
C_max_ (μg/L)	3015.8 ± 1076.7	4899.4 ± 2521.8	5037.5 ± 1437.8	2953.4 ± 848.1
Total CV (%)	35.7	51.5	28.5	28.7
AUC_last_ (h·μg/L)	55,676.6 ± 13,708.3	89,241.2 ± 30,037.6	105,312.9 ± 21,297.7	73,730.1 ± 28,864.1
Total CV (%)	24.6	33.7	20.2	39.2
T_max_ (h)	4.0 [2.0–10.0]	4.0 [2.0–4.0]	4.0 [2.0–10.0]	4.0 [3.0–10.0]
t_1/2_ (h)	20.9 ± 4.2	24.7 ± 8.6	24.4 ± 7.2	22.2 ± 5.1

^†^ Mean ± standard deviation is presented for C_max_, AUC_last_ and t_1/2_. The median [minimum–maximum] value is presented for T_max_. Abbreviations: C_max_, maximum plasma concentration; CV, coefficient of variation; GMR, geometric mean ratio; AUC_last_, area under the time-concentration curve from dosing to the last measurable concentration; T_max_, time to reach C_max_; t_1/2_, terminal elimination half-life.

**Table 2:** Parameter estimates of the final model and bootstrap result.

Parameters	Parameter Estimates (Relative Standard Error, %)	Median of Bootstrap Results (95% Confidence Intervals)
Relative bioavailability ^†^	1.62 (8.7)	1.62 (1.34–1.91)
MTT (h)	3.24 (5.4)	3.24 (2.89–3.59)
NN	1.72 (7.3)	1.75 (1.46–1.98)
k_a_ (h^−1^)	1.6 (16.8)	1.68 (0.79–2.41)
CL (L/h)	3.33 (9.6)	3.23 (2.6–4.06)
V (L)	8.17 (31.5)	8.35 (3.16–13.19)
F_ent_	0.215 (13.5)	0.19 (0.13–0.3)
k_Ehc_ (h^−1^)	0.212 (6.8)	0.21 (0.18–0.24)
T_gb1_ (h)	8.14 (0.3)	8.14 (8.1–8.19)
T_gb2_ (h)	12.2 (0.5)	12.16 (12.04–12.29)
IIV k_a_ (%CV)	87.3 (17.0)	90.5 (55.4–138.9)
IIV CL (%CV)	26.1 (13.2)	25.0 (17.7–31.8)
IIV V (%CV)	21.5 (17.1)	21.0 (13.2–28.2)
IOV CL (%CV)	9.0 (16.4)	9.0 (5.9–11.8)
Proportional residual error (%CV)	37.6 (4.4)	36.1 (33.3–39.5)

^†^ SYO-1644 to Nexavar^®^. Abbreviations: MTT, mean transit time; NN, number of transit compartments; ka, absorption rate constant; CL, clearance of the central compartment; V, volume of distribution of the central compartment; F_ent_, fraction of enterohepatic recirculation; k_Ehc_, enterohepatic recirculation constant; T_gb1_, early gall bladder emptying time; T_gb2_, late gall bladder emptying time; IIV, inter-individual variability; IOV, inter-occasional variability; CV, coefficient of variation.

## Data Availability

The data presented in this study are available upon request from the corresponding author. The data were not publicly available because of confidentiality.

## Supplementary Materials

The following are available online at https://www.mdpi.com/article/10.3390/pharmaceutics13050629/s1, Figure S1: goodness-of-fit plots., Table S1: summary of objective function values for the evaluated structure models, Table S2: proportion and its 95% confidence interval of bioequivalence achievement based on simulated 2 × 2 bioequivalence trials for 500 times according to SYO-1644 dose and the number of subjects in each trial, Table S3: summary of intra-coefficient of variation of C_max_ and AUC_last_ in simulated trials according to SYO-1644 dose and the number of subjects in each trial.
