# Supplementary material for: Population Pharmacokinetic Modelling and Simulation to Determine the Optimal Dose of Nanoparticulated Sorafenib to the Reference Sorafenib
Source: Pharmaceutics. 2021 Apr 28;13(5):629. doi: 10.3390/pharmaceutics13050629 (PMC8145937; doi:10.3390/pharmaceutics13050629)
Supplement: Supplementary file 1 [file pharmaceutics-13-00629-s001.zip › pharmaceutics-1181964-supplementary.pdf]

pharmaceutics

Supplementary Materials: Population Pharmacokinetic Modelling and Simulation to Determine the Optimal Dose of Nanoparticulated Sorafenib to the Reference Sorafenib

Ki Young Huh, Sejung Hwang, Sang Yeob Park, Hye Jung Lim, Miryung Jin [2], Jaeseong Oh, Kyung–Sang Yu and Jae–Yong Chung

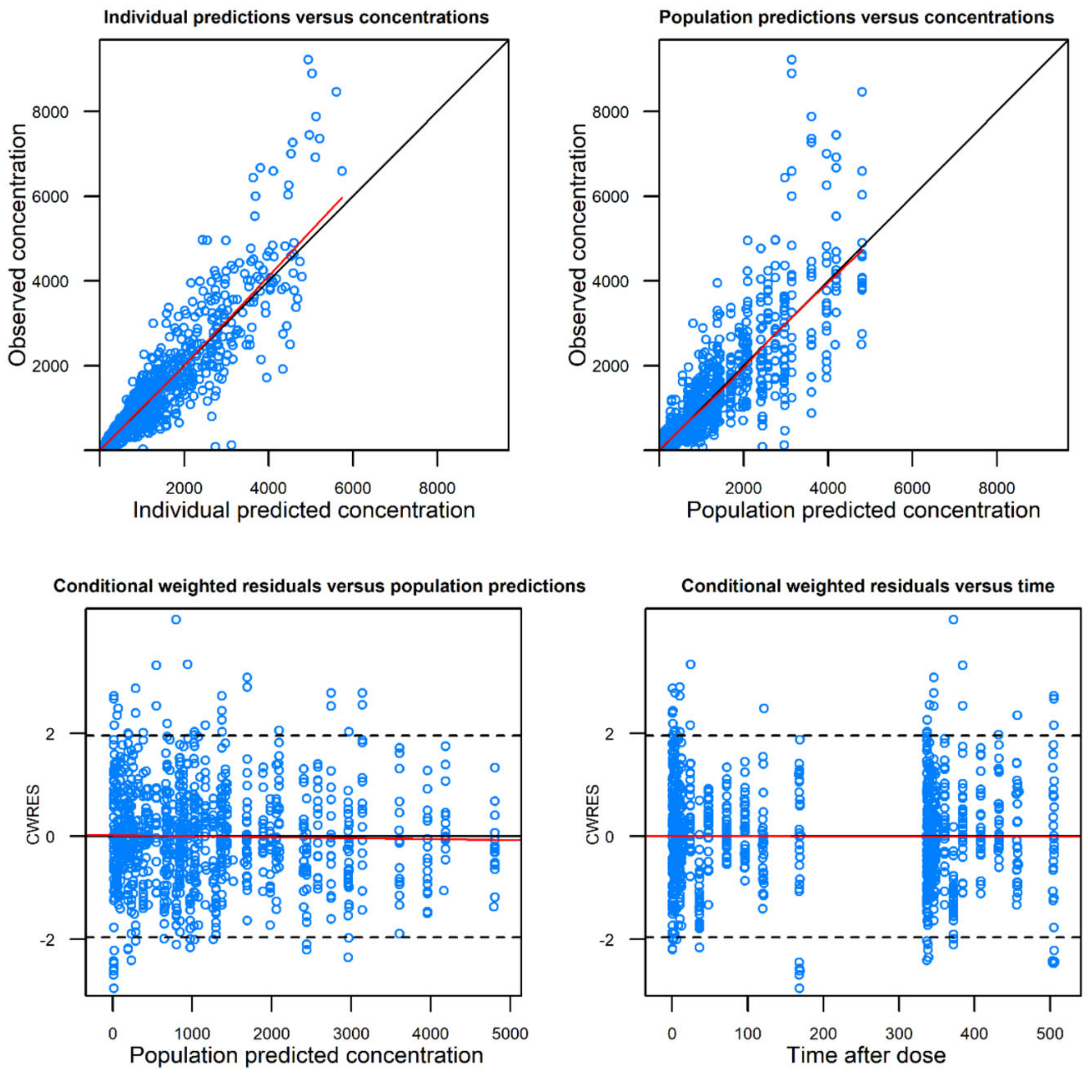

Figure S1. Goodness–of–fit plots.

Table 1. Summary of objective function values for the evaluated structure models.

Model Description	Objective Function Values	Comments
Zero and first order combined absorption	16,230	Not satisfactory goodness of fit
Zero and first order combined absorption, IIV on CL	11,288	Not satisfactory goodness of fit
Zero and first order combined absorption, IIV on CL, V2	10,088	Not satisfactory goodness of fit
Zero and first order combined absorption with a lag time on compartment 1, IIV on CL, V2	10,050	Not satisfactory goodness of fit
Enterohepatic reabsorption model with bile compartment (continuous release)	11,934	
Enterohepatic reabsorption model with bile compartment, IIV on CL	11,667	
Enterohepatic reabsorption model with bile compartment, IIV on CL, V2	11,558	
Enterohepatic reabsorption model with bile compartment, IIV on CL, V2, transition time from compartment 3 to 4	11.548	
Enterohepatic reabsorption model with bile compartment connected to depot compartment (continuous release)	11,957	
Enterohepatic reabsorption model with bile compartment connected to depot compartment, IIV on CL	11,657	
Enterohepatic reabsorption model with bile compartment connected to depot compartment, IIV on CL, V2	11,547	
Enterohepatic reabsorption model with bile compartment connected to depot compartment, IIV on CL, V2, transition time from compartment 3 to 4	11,536	
Enterohepatic reabsorption model with transit compartments	11,346	
Enterohepatic reabsorption model with transit compartments, IIV on CL	11,005	
Enterohepatic reabsorption model with transit compartments, IIV on CL, V2	10,871	
Enterohepatic reabsorption model with transit compartments, IIV on CL, V2, KA	10,767	
Enterohepatic reabsorption model with transit compartments, IIV on CL, V2, KA, IOV on CL, with sigmoid function	10,735	
Enterohepatic reabsorption model with transit compartments, with sigmoid and switch function with early and late gall bladder secretion time, IIV on CL, V2, KA, IOV on CL (final model)	10,604	

Notes: CL, clearance; IIV, inter-individual variability; IOV, inter-occasional variability; V2, volume of distribution of the second compartment

Table S2. Proportion and its 95% confidence interval of bioequivalence achievement based on simulated 2 × 2 bioequivalence trials for 500 times according to SYO–1644 dose and the number of subjects in each trial.

**Dose**	**Number of Subjects**												
	**24**	**26**	**28**	**30**	**32**	**34**	**36**	**38**	**40**	**42**	**44**	**46**	**48**
Proportion and 95% confidence intervals of bioequivalence achievement of C_{max}													
100	0.05 (0.03–0.07)	0.06 (0.04–0.08)	0.07 (0.05–0.09)	0.06 (0.04–0.09)	0.08 (0.05–0.10)	0.08 (0.05–0.10)	0.07 (0.05–0.09)	0.07 (0.04–0.09)	0.08 (0.06–0.10)	0.09 (0.06–0.12)	0.07 (0.04–0.09)	0.06 (0.04–0.08)	0.08 (0.06–0.10)
105	0.23 (0.19–0.27)	0.25 (0.21–0.29)	0.25 (0.21–0.29)	0.27 (0.23–0.31)	0.29 (0.25–0.33)	0.29 (0.25–0.33)	0.31 (0.27–0.35)	0.30 (0.26–0.34)	0.32 (0.28–0.36)	0.33 (0.29–0.38)	0.32 (0.28–0.36)	0.35 (0.31–0.39)	0.35 (0.31–0.39)
110	0.46 (0.42–0.51)	0.51 (0.47–0.55)	0.54 (0.49–0.58)	0.55 (0.51–0.59)	0.58 (0.54–0.63)	0.61 (0.57–0.65)	0.63 (0.58–0.67)	0.67 (0.63–0.71)	0.67 (0.63–0.71)	0.65 (0.61–0.69)	0.70 (0.66–0.74)	0.71 (0.67–0.75)	0.71 (0.67–0.75)
115	0.74 (0.70–0.78)	0.77 (0.74–0.81)	0.79 (0.75–0.82)	0.82 (0.79–0.85)	0.83 (0.80–0.87)	0.85 (0.81–0.88)	0.88 (0.85–0.90)	0.88 (0.85–0.91)	0.90 (0.87–0.92)	0.91 (0.89–0.94)	0.92 (0.89–0.94)	0.93 (0.90–0.95)	0.94 (0.92–0.96)
120	0.88 (0.85–0.91)	0.91 (0.88–0.93)	0.91 (0.89–0.94)	0.94 (0.92–0.96)	0.95 (0.94–0.97)	0.97 (0.96–0.98)	0.98 (0.96–0.99)	0.98 (0.97–0.99)	0.99 (0.98–1.00)	0.99 (0.98–1.00)	0.99 (0.99–1.00)	1.00 (0.99–1.00)	1.00 (0.99–1.00)
125	0.92 (0.89–0.94)	0.93 (0.91–0.95)	0.93 (0.91–0.95)	0.96 (0.95–0.98)	0.98 (0.97–0.99)	0.98 (0.97–0.99)	0.98 (0.97–0.99)	0.99 (0.99–1.00)	1.00 (0.99–1.00)	1.00 (0.99–1.00)	1.00 (0.99–1.00)	1.00 (0.99–1.00)	1.00 (1.00–1.00)
130	0.82 (0.79–0.86)	0.85 (0.82–0.88)	0.88 (0.86–0.91)	0.90 (0.88–0.93)	0.92 (0.89–0.94)	0.93 (0.91–0.95)	0.94 (0.92–0.96)	0.95 (0.94–0.97)	0.97 (0.95–0.98)	0.98 (0.96–0.99)	0.97 (0.96–0.99)	0.98 (0.97–0.99)	0.98 (0.97–0.99)
135	0.66 (0.62–0.71)	0.72 (0.68–0.76)	0.74 (0.70–0.78)	0.76 (0.72–0.79)	0.76 (0.73–0.80)	0.80 (0.76–0.83)	0.82 (0.79–0.86)	0.85 (0.81–0.88)	0.86 (0.83–0.89)	0.87 (0.84–0.90)	0.89 (0.86–0.92)	0.90 (0.88–0.93)	0.90 (0.87–0.92)
140	0.45 (0.41–0.50)	0.49 (0.44–0.53)	0.53 (0.48–0.57)	0.54 (0.49–0.58)	0.57 (0.52–0.61)	0.57 (0.52–0.61)	0.61 (0.57–0.65)	0.63 (0.59–0.68)	0.65 (0.61–0.69)	0.65 (0.61–0.70)	0.69 (0.65–0.73)	0.71 (0.67–0.75)	0.73 (0.69–0.77)
145	0.26 (0.22–0.30)	0.27 (0.23–0.31)	0.31 (0.27–0.35)	0.29 (0.25–0.33)	0.32 (0.28–0.36)	0.34 (0.30–0.38)	0.36 (0.31–0.40)	0.33 (0.29–0.37)	0.36 (0.31–0.40)	0.39 (0.34–0.43)	0.41 (0.36–0.45)	0.41 (0.37–0.46)	0.42 (0.37–0.46)
150	0.14 (0.11–0.17)	0.11 (0.08–0.14)	0.12 (0.10–0.15)	0.14 (0.11–0.17)	0.13 (0.10–0.16)	0.16 (0.12–0.19)	0.16 (0.13–0.19)	0.16 (0.13–0.19)	0.17 (0.14–0.21)	0.17 (0.14–0.20)	0.15 (0.12–0.19)	0.17 (0.14–0.20)	0.18 (0.15–0.22)
Proportion and 95% confidence intervals of bioequivalence achievement of AUC_{last}													
100	0.07 (0.05–0.10)	0.07 (0.05–0.09)	0.07 (0.04–0.09)	0.09 (0.06–0.11)	0.08 (0.06–0.10)	0.09 (0.07–0.12)	0.10 (0.07–0.12)	0.08 (0.06–0.10)	0.09 (0.07–0.12)	0.08 (0.05–0.10)	0.09 (0.06–0.12)	0.09 (0.06–0.11)	0.10 (0.08–0.13)
105	0.35 (0.31–0.40)	0.39 (0.34–0.43)	0.41 (0.36–0.45)	0.41 (0.37–0.46)	0.43 (0.39–0.48)	0.46 (0.42–0.51)	0.48 (0.44–0.53)	0.48 (0.43–0.52)	0.49 (0.45–0.54)	0.54 (0.49–0.58)	0.53 (0.48–0.57)	0.53 (0.49–0.57)	0.56 (0.51–0.60)

110	0.72 (0.68–0.76)	0.76 (0.72–0.80)	0.81 (0.78–0.84)	0.83 (0.80–0.86)	0.83 (0.80–0.87)	0.82 (0.79–0.85)	0.88 (0.85–0.91)	0.88 (0.85–0.91)	0.90 (0.87–0.92)	0.91 (0.88–0.94)	0.92 (0.90–0.94)	0.92 (0.90–0.94)	0.94 (0.91–0.96)
115	0.94 (0.92–0.96)	0.97 (0.96–0.99)	0.97 (0.95–0.98)	0.98 (0.97–0.99)	0.98 (0.97–0.99)	0.99 (0.98–1.00)	0.99 (0.98–1.00)	0.99 (0.98–1.00)	0.99 (0.98–1.00)	0.99 (0.99–1.00)	1.00 (0.99–1.00)	1.00 (1.00–1.00)	1.00 (0.99–1.00)
120	0.99 (0.99–1.00)	1.00 (0.99–1.00)	1.00 (0.99–1.00)	1.00 (1.00–1.00)	1.00 (1.00–1.00)	1.00 (0.99–1.00)	1.00 (1.00–1.00)	1.00 (1.00–1.00)	1.00 (1.00–1.00)	1.00 (1.00–1.00)	1.00 (1.00–1.00)	1.00 (1.00–1.00)	1.00 (1.00–1.00)
125	1.00 (0.99–1.00)	1.00 (0.99–1.00)	1.00 (1.00–1.00)	1.00 (1.00–1.00)	1.00 (0.99–1.00)	1.00 (1.00–1.00)	1.00 (1.00–1.00)	1.00 (1.00–1.00)	1.00 (1.00–1.00)	1.00 (1.00–1.00)	1.00 (1.00–1.00)	1.00 (1.00–1.00)	1.00 (1.00–1.00)
130	0.97 (0.96–0.99)	0.99 (0.98–1.00)	0.99 (0.98–1.00)	0.99 (0.99–1.00)	0.99 (0.99–1.00)	0.99 (0.98–1.00)	1.00 (0.99–1.00)	1.00 (0.99–1.00)	1.00 (0.99–1.00)	1.00 (0.99–1.00)	1.00 (1.00–1.00)	1.00 (0.99–1.00)	1.00 (0.99–1.00)
135	0.91 (0.89–0.94)	0.93 (0.91–0.95)	0.95 (0.93–0.97)	0.95 (0.93–0.97)	0.96 (0.94–0.97)	0.96 (0.95–0.98)	0.98 (0.96–0.99)	0.98 (0.97–0.99)	0.99 (0.98–1.00)	0.98 (0.97–0.99)	0.99 (0.98–1.00)	0.99 (0.98–1.00)	0.99 (0.99–1.00)
140	0.70 (0.66–0.74)	0.73 (0.69–0.77)	0.76 (0.72–0.80)	0.78 (0.74–0.81)	0.79 (0.75–0.82)	0.82 (0.79–0.86)	0.85 (0.82–0.88)	0.87 (0.84–0.90)	0.87 (0.84–0.90)	0.89 (0.87–0.92)	0.89 (0.86–0.92)	0.92 (0.89–0.94)	0.92 (0.89–0.94)
145	0.38 (0.34–0.42)	0.39 (0.35–0.44)	0.44 (0.39–0.48)	0.49 (0.44–0.53)	0.47 (0.42–0.51)	0.50 (0.46–0.55)	0.52 (0.48–0.57)	0.56 (0.52–0.61)	0.57 (0.53–0.62)	0.58 (0.54–0.63)	0.60 (0.56–0.64)	0.62 (0.58–0.66)	0.65 (0.61–0.69)
150	0.15 (0.12–0.18)	0.15 (0.12–0.18)	0.14 (0.11–0.17)	0.16 (0.13–0.19)	0.18 (0.14–0.21)	0.21 (0.17–0.24)	0.20 (0.17–0.24)	0.19 (0.16–0.22)	0.22 (0.18–0.25)	0.21 (0.17–0.24)	0.23 (0.20–0.27)	0.22 (0.19–0.26)	0.25 (0.21–0.29)

Table S3. Summary of intra–coefficient of variation of C_{max} and AUC_{last} in simulated trials according to SYO–1644 dose and the number of subjects in each trial.

**Dose**	**Number of Subjects**												
	**24**	**26**	**28**	**30**	**32**	**34**	**36**	**38**	**40**	**42**	**44**	**46**	**48**
Intra–coefficient of variation of C_{max} (%, Median [minimum–maximum])													
100	22 [13.6–32.2]	22.2 [13.1–32.5]	22.1 [13.9–32.3]	22.2 [13.1–32.9]	22 [15–33]	22.1 [15–34.2]	22.1 [15.1–30.5]	22.2 [15.7–31.4]	22.2 [15.8–30.3]	22.1 [15.2–32.2]	22.3 [15.5–31.6]	22.2 [16.2–29.4]	22.2 [15.4–30.6]
105	22 [13.6–32.2]	22.2 [13.1–32.5]	22.1 [13.9–32.3]	22.2 [13.1–32.9]	22 [15–33]	22.1 [15–34.2]	22.1 [15.1–30.5]	22.2 [15.7–31.4]	22.2 [15.8–30.3]	22.1 [15.2–32.2]	22.3 [15.5–31.6]	22.2 [16.2–29.4]	22.2 [15.4–30.6]
110	22 [13.6–32.2]	22.2 [13.1–32.5]	22.1 [13.9–32.3]	22.2 [13.1–32.9]	22 [15–33]	22.1 [15–34.2]	22.1 [15.1–30.5]	22.2 [15.7–31.4]	22.2 [15.8–30.3]	22.1 [15.2–32.2]	22.3 [15.5–31.6]	22.2 [16.2–29.4]	22.2 [15.4–30.6]
115	22 [13.6–32.2]	22.2 [13.1–32.5]	22.1 [13.9–32.3]	22.2 [13.1–32.9]	22 [15–33]	22.1 [15–34.2]	22.1 [15.1–30.5]	22.2 [15.7–31.4]	22.2 [15.8–30.3]	22.1 [15.2–32.2]	22.3 [15.5–31.6]	22.2 [16.2–29.4]	22.2 [15.4–30.6]
120	22 [13.6–32.2]	22.2 [13.1–32.5]	22.1 [13.9–32.3]	22.2 [13.1–32.9]	22 [15–33]	22.1 [15–34.2]	22.1 [15.1–30.5]	22.2 [15.7–31.4]	22.2 [15.8–30.3]	22.1 [15.2–32.2]	22.3 [15.5–31.6]	22.2 [16.2–29.4]	22.2 [15.4–30.6]
125	22 [13.6–32.2]	22.2 [13.1–32.5]	22.1 [13.9–32.3]	22.2 [13.1–32.9]	22 [15–33]	22.1 [15–34.2]	22.1 [15.1–30.5]	22.2 [15.7–31.4]	22.2 [15.8–30.3]	22.1 [15.2–32.2]	22.3 [15.5–31.6]	22.2 [16.2–29.4]	22.2 [15.4–30.6]
130	22 [13.6–32.2]	22.2 [13.1–32.5]	22.1 [13.9–32.3]	22.2 [13.1–32.9]	22 [15–33]	22.1 [15–34.2]	22.1 [15.1–30.5]	22.2 [15.7–31.4]	22.2 [15.8–30.3]	22.1 [15.2–32.2]	22.3 [15.5–31.6]	22.2 [16.2–29.4]	22.2 [15.4–30.6]
135	22 [13.6–32.2]	22.2 [13.1–32.5]	22.1 [13.9–32.3]	22.2 [13.1–32.9]	22 [15–33]	22.1 [15–34.2]	22.1 [15.1–30.5]	22.2 [15.7–31.4]	22.2 [15.8–30.3]	22.1 [15.2–32.2]	22.3 [15.5–31.6]	22.2 [16.2–29.4]	22.2 [15.4–30.6]
140	22 [13.6–32.2]	22.2 [13.1–32.5]	22.1 [13.9–32.3]	22.2 [13.1–32.9]	22 [15–33]	22.1 [15–34.2]	22.1 [15.1–30.5]	22.2 [15.7–31.4]	22.2 [15.8–30.3]	22.1 [15.2–32.2]	22.3 [15.5–31.6]	22.2 [16.2–29.4]	22.2 [15.4–30.6]
145	22 [13.6–32.2]	22.2 [13.1–32.5]	22.1 [13.9–32.3]	22.2 [13.1–32.9]	22 [15–33]	22.1 [15–34.2]	22.1 [15.1–30.5]	22.2 [15.7–31.4]	22.2 [15.8–30.3]	22.1 [15.2–32.2]	22.3 [15.5–31.6]	22.2 [16.2–29.4]	22.2 [15.4–30.6]
150	22 [13.6–32.2]	22.2 [13.1–32.5]	22.1 [13.9–32.3]	22.2 [13.1–32.9]	22 [15–33]	22.1 [15–34.2]	22.1 [15.1–30.5]	22.2 [15.7–31.4]	22.2 [15.8–30.3]	22.1 [15.2–32.2]	22.3 [15.5–31.6]	22.2 [16.2–29.4]	22.2 [15.4–30.6]
Intra–coefficient of variation of AUC_{last} (%, Median [minimum–maximum])													
100	15.7 [8.4–22.2]	15.5 [9.6–22.2]	15.5 [9.8–22.7]	15.5 [10.8–22.7]	15.6 [10.3–22.8]	15.7 [10.3–20.8]	15.5 [9.9–22.5]	15.6 [11.1–21.3]	15.5 [10.6–21.1]	15.6 [11.1–21.3]	15.4 [10.8–21.3]	15.7 [11.3–21.3]	15.6 [11.3–20.3]
105	15.7 [8.4–22.2]	15.5 [9.6–22.2]	15.5 [9.8–22.7]	15.5 [10.8–22.7]	15.6 [10.3–22.8]	15.7 [10.3–20.8]	15.5 [9.9–22.5]	15.6 [11.1–21.3]	15.5 [10.6–21.1]	15.6 [11.1–21.3]	15.4 [10.8–21.3]	15.7 [11.3–21.3]	15.6 [11.3–20.3]
110	15.7	15.5	15.5	15.5	15.6	15.7	15.5	15.6	15.5	15.6	15.4	15.7	15.6

	[8.4–22.2]	[9.6–22.2]	[9.8–22.7]	[10.8–22.7]	[10.3–22.8]	[10.3–20.8]	[9.9–22.5]	[11.1–21.3]	[10.6–21.1]	[11.1–21.3]	[10.8–21.3]	[11.3–21.3]	[11.3–20.3]
115	15.7	15.5	15.5	15.5	15.6	15.7	15.5	15.6	15.5	15.6	15.4	15.7	15.6
	[8.4–22.2]	[9.6–22.2]	[9.8–22.7]	[10.8–22.7]	[10.3–22.8]	[10.3–20.8]	[9.9–22.5]	[11.1–21.3]	[10.6–21.1]	[11.1–21.3]	[10.8–21.3]	[11.3–21.3]	[11.3–20.3]
120	15.7	15.5	15.5	15.5	15.6	15.7	15.5	15.6	15.5	15.6	15.4	15.7	15.6
	[8.4–22.2]	[9.6–22.2]	[9.8–22.7]	[10.8–22.7]	[10.3–22.8]	[10.3–20.8]	[9.9–22.5]	[11.1–21.3]	[10.6–21.1]	[11.1–21.3]	[10.8–21.3]	[11.3–21.3]	[11.3–20.3]
125	15.7	15.5	15.5	15.5	15.6	15.7	15.5	15.6	15.5	15.6	15.4	15.7	15.6
	[8.4–22.2]	[9.6–22.2]	[9.8–22.7]	[10.8–22.7]	[10.3–22.8]	[10.3–20.8]	[9.9–22.5]	[11.1–21.3]	[10.6–21.1]	[11.1–21.3]	[10.8–21.3]	[11.3–21.3]	[11.3–20.3]
130	15.7	15.5	15.5	15.5	15.6	15.7	15.5	15.6	15.5	15.6	15.4	15.7	15.6
	[8.4–22.2]	[9.6–22.2]	[9.8–22.7]	[10.8–22.7]	[10.3–22.8]	[10.3–20.8]	[9.9–22.5]	[11.1–21.3]	[10.6–21.1]	[11.1–21.3]	[10.8–21.3]	[11.3–21.3]	[11.3–20.3]
135	15.7	15.5	15.5	15.5	15.6	15.7	15.5	15.6	15.5	15.6	15.4	15.7	15.6
	[8.4–22.2]	[9.6–22.2]	[9.8–22.7]	[10.8–22.7]	[10.3–22.8]	[10.3–20.8]	[9.9–22.5]	[11.1–21.3]	[10.6–21.1]	[11.1–21.3]	[10.8–21.3]	[11.3–21.3]	[11.3–20.3]
140	15.7	15.5	15.5	15.5	15.6	15.7	15.5	15.6	15.5	15.6	15.4	15.7	15.6
	[8.4–22.2]	[9.6–22.2]	[9.8–22.7]	[10.8–22.7]	[10.3–22.8]	[10.3–20.8]	[9.9–22.5]	[11.1–21.3]	[10.6–21.1]	[11.1–21.3]	[10.8–21.3]	[11.3–21.3]	[11.3–20.3]
145	15.7	15.5	15.5	15.5	15.6	15.7	15.5	15.6	15.5	15.6	15.4	15.7	15.6
	[8.4–22.2]	[9.6–22.2]	[9.8–22.7]	[10.8–22.7]	[10.3–22.8]	[10.3–20.8]	[9.9–22.5]	[11.1–21.3]	[10.6–21.1]	[11.1–21.3]	[10.8–21.3]	[11.3–21.3]	[11.3–20.3]
150	15.7	15.5	15.5	15.5	15.6	15.7	15.5	15.6	15.5	15.6	15.4	15.7	15.6
	[8.4–22.2]	[9.6–22.2]	[9.8–22.7]	[10.8–22.7]	[10.3–22.8]	[10.3–20.8]	[9.9–22.5]	[11.1–21.3]	[10.6–21.1]	[11.1–21.3]	[10.8–21.3]	[11.3–21.3]	[11.3–20.3]

Table S3. Summary of objective function values for the evaluated structure models.

Model Description	Objective Function Values	Comments
Zero and first order combined absorption	16,230	Not satisfactory goodness of fit
Zero and first order combined absorption, IIV on CL	11,288	Not satisfactory goodness of fit
Zero and first order combined absorption, IIV on CL, V2	10,088	Not satisfactory goodness of fit
Zero and first order combined absorption with a lag time on compartment 1, IIV on CL, V2	10,050	Not satisfactory goodness of fit
Enterohepatic reabsorption model with bile compartment (continuous release)	11,934	
Enterohepatic reabsorption model with bile compartment, IIV on CL	11,667	
Enterohepatic reabsorption model with bile compartment, IIV on CL, V2	11,558	
Enterohepatic reabsorption model with bile compartment, IIV on CL, V2, transition time from compartment 3 to 4	11.548	

Enterohepatic reabsorption model with bile compartment connected to depot compartment (continuous release)	11,957
Enterohepatic reabsorption model with bile compartment connected to depot compartment, IIV on CL	11,657
Enterohepatic reabsorption model with bile compartment connected to depot compartment, IIV on CL, V2	11,547
Enterohepatic reabsorption model with bile compartment connected to depot compartment, IIV on CL, V2, transition time from compartment 3 to 4	11,536
Enterohepatic reabsorption model with transit compartments	11,346
Enterohepatic reabsorption model with transit compartments, IIV on CL	11,005
Enterohepatic reabsorption model with transit compartments, IIV on CL, V2	10,871
Enterohepatic reabsorption model with transit compartments, IIV on CL, V2, KA	10,767
Enterohepatic reabsorption model with transit compartments, IIV on CL, V2, KA, IOV on CL, with sigmoid function	10,735
Enterohepatic reabsorption model with transit compartments, with sigmoid and switch function with early and late gall bladder secretion time, IIV on CL, V2, KA, IOV on CL (final model)	10,604

Notes: CL, clearance; IIV, inter-individual variability; IOV, inter-occasional variability; V2, volume of distribution of the second compartm
